# ZnO@C (core@shell) microspheres derived from spent coffee grounds as applicable non-precious electrode material for DMFCs

**DOI:** 10.1038/s41598-017-01463-3

**Published:** 2017-05-11

**Authors:** Zafar Khan Ghouri, Saeed Al-Meer, Nasser A. M. Barakat, Hak Yong Kim

**Affiliations:** 1 Central Laboratory Unit, Qatar University, P. O. Box: 2713 Doha, Qatar; 2 Chemical Engineering Department, Faculty of Engineering, El-Minia University, El-Minia, Egypt; 3 Organic Material and Fiber Engineering Department, Chonbuk National University, Jeonju, 561-756 Republic of Korea; 4 Department of BIN Convergence Technology, Chonbuk National University, Jeonju, 561-756 Republic of Korea

## Abstract

Although numerous reports have introduced non precious electrocatalysts for methanol oxidation, most of those studies did not consider the corresponding high onset potential which restricts utilization in real fuel cells. In this study, an −90 mV [vs. Ag/AgCl] onset potential non-precious electrocatalyst is introduced as an applicable anode material for the direct methanol fuel cells. Moreover, the proposed material was prepared from a cheap and abundantly existing resource; the spent coffee grounds. Typically, the spent coffee grounds were facilely converted to core@shell (ZnO@C) microspheres through a two-step approach, involving chemical activation and a subsequent calcination at temperature of 700 °C. Activation of the carbon derived from the spent coffee grounds was performed with ZnCl_2_ which acts as pore-forming agent as well as a precursor for the ZnO. The structure and morphology were characterized by (XRD), (SEM), and (TEM) analyses while the electrochemical characterizations was evaluated by cyclic voltammetry (CV) technique. Besides the comparatively very low onset potential, the introduced microspheres exhibited relatively high current density; 17 mA/cm^2^. Overall, based on the advantages of the green source of carbon and the good electrocatalytic activity, the spent coffee grounds-derived carbon can be considered a promise anode material for the DMFCs.

## Introduction

Owing to the increase in the energy demand, near expected exhaustion of the fossil oil resources and environment concerns, developing of an alternate resource of energy is highly considered an essential requirement^1–3^. In response, fuel cell is a respectable technological choice as a green energy source for solving energy and pollution problems owing many advantages such as spontaneous conversion of the chemical energy to electrical energy through the process of electrochemical oxidation, low pollution and environmental friendliness^4–7^. Among the various types of fuel cells, direct methanol fuel cells (DMFCs) recently received much attention by the research and development communities due to the easy storage and transportation of fuels, excellent efficiency at low operating temperature and system simplicity^4, 5, 8^. The efficiency of the fuel cell is directly related to the catalytic activity of the electrode material; Pt-based electrocatalysts are considered the standard anode materials for the DMFCs^9–12^.

However, too high production cost, difficulties in supply chain, carbon monoxide (CO) adsorbate poisoning and the insufficient durability of the widely used Pt-based catalysts are the main drawbacks for the commercialization of the DMFCs^4, 5^. In literature, there are numerous studies introduced non-precious electrocatalysts to replace Pt-based electrodes; most of them are transition metals. Basically, to be applicable in a real fuel cell, the anode potential should be lower than the cathode one. As the commercial fuel cells are based on air-cathodes, in alkaline medium, the corresponding potential of the oxygen reduction reaction (ORR) is around 0.44 V (vs. NHE and 0.24 vs. Ag/AgCl). Accordingly, the onset potential of any introduced anode material for the DMFCs should be lower than the aforementioned oxygen reduction reaction potential. Unfortunately, most of the introduced anode materials (especially nickel-based ones which are the most widely reported) do not own this important characteristic. Accordingly, based on our best knowledge, the non-precious electrocatalysts were not commercially utilized yet. The most widely studied non-precious metals are Ni, Co, Cu, Ce, and their alloys and oxides^4, 5, 13–15^.

On the other hand, to overcome the high cost of the precious metals, support materials were exploited for the development of highly efficient and relatively cheap electrocatalysts. Carbon supports have shown distinct enhancement in the electrocatalytic activity for both of the precious and non-precious functional materials due to the high adsorption affinity^8, 16–18^.

Although, many researchers ignored ZnO to be utilized as catalyst in the fuel cell applications, recent reports have proved that this metal demonstrates both semiconducting and piezoelectric characteristics simultaneously which may enhance its catalytic activity^19^. Moreover, it was proved that the anodic reactions in the DMFCs can be well-thought-out as a combination of adsorption and electrochemical reaction on the anode surface^20–22^.

Our work aims to develop industrially-applicable and efficient non-precious electrocatalyst for methanol oxidation from a cheap and abundantly existing resource; spent coffee grounds. In the present study, activation of carbon-derived from spent coffee grounds was performed with ZnCl_2_, where ZnCl_2_ acts as a pore-forming agent as well as a precursor for ZnO. The coffee is the most highly consumed liquid refreshment. As high amount of leftover is being produced, struggles have been made to reuse the consumed coffee grounds for energy production^23, 24^. Herein, spent coffee grounds was facilely converted to core@shell (ZnO@C) microspheres through a two-step approach, involving a chemical activation and a subsequent calcination at 700 °C. The promising textural property of the core@shell microspheres led to create a distinct catalytic activity for methanol electro oxidation in the alkaline medium which was reflected in very low onset potential and acceptable current density.

## Results and Discussion

**Figure 1:**
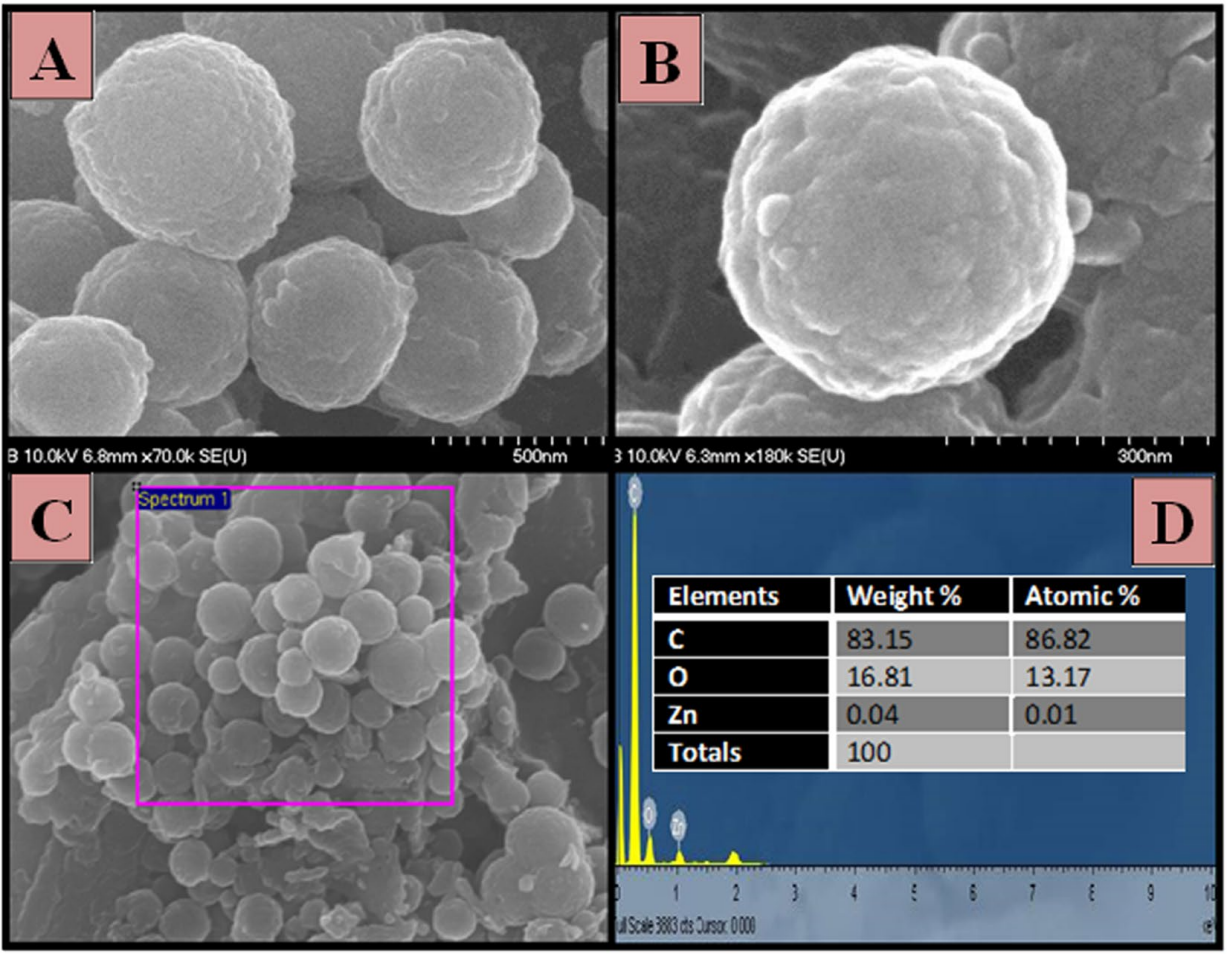
(**A**) FESEM image, (**B**) High resolution FESEM image, (**C & D**) SEM images with corresponding EDS maps for Zn, C and O of produced core@shell (ZnO@C) microspheres after carbonized at 700 °C for 2 h in nitrogen atmosphere.

The morphology and structure of the produced powder after the calcination process were explored by scanning electron microscopy (SEM). As seen, microspheres were obtained with a mean diameter of 500 nm (Fig. 1(A)); the magnified image (Fig. 1(B)) shows that the carbon spheres are significantly rough and possessed well defined spherical structure. A particular area of the EDX spectrum from Fig. 1(C) was taken, and the results are established in Fig. 1(D). The EDS spectrum displays a clear signal of Zn and O besides C signals, no other peaks were detected in the range which clearly confirmed that the microspheres are purely made of Zn, O, and C. The atomic elemental percentage of carbon, oxygen and Zinc are summarized in the inset in Fig. 1(D).

**Figure 2:**
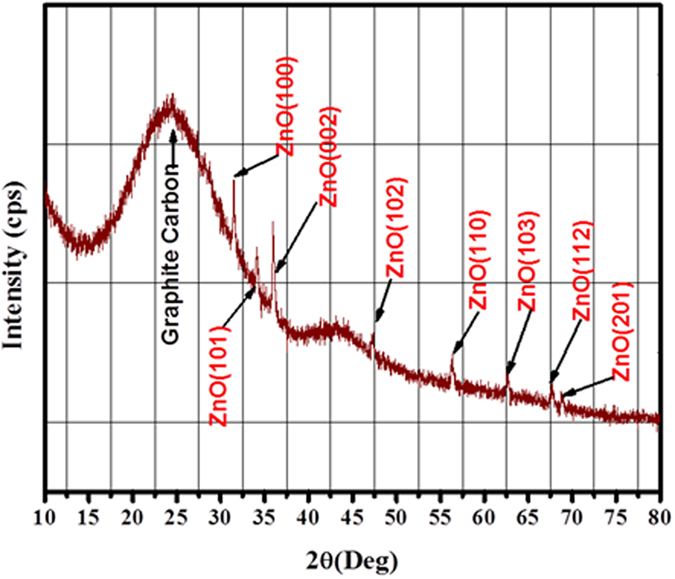
XRD pattern for the obtained core@shell (ZnO@C) microspheres after carbonized at 700 °C for 2 h in nitrogen atmosphere.

The obtained microspheres were subjected to XRD investigation. The X–ray diffraction pattern (Fig. 2) exhibited sharp intense peaks at 2θ values of 31.69°, 34.38°, 36.18°, 47.45°, 56.46°, 62.76°, 67.80° and 68.92° corresponding to (100), (002), (101), (102), (110), (103), (112) and (201) crystal planes, respectively indexed to the hexagonal structure of ZnO (Ref. Code: 01–079–0207) with lattice constants a = b = 3.2568 and c = 5.2125 Å; α = β = 90° and γ = 120° [space group: P63mc]. No diffraction peaks corresponding to the used precursor (ZnCl_2_) were detected in the XRD pattern indicating that the sample contains high phase purity of ZnO. Furthermore, a broad peak appears near 2θ = 25° which is consistent with the (002) reflection for the graphitic carbon.

**Figure 3:**
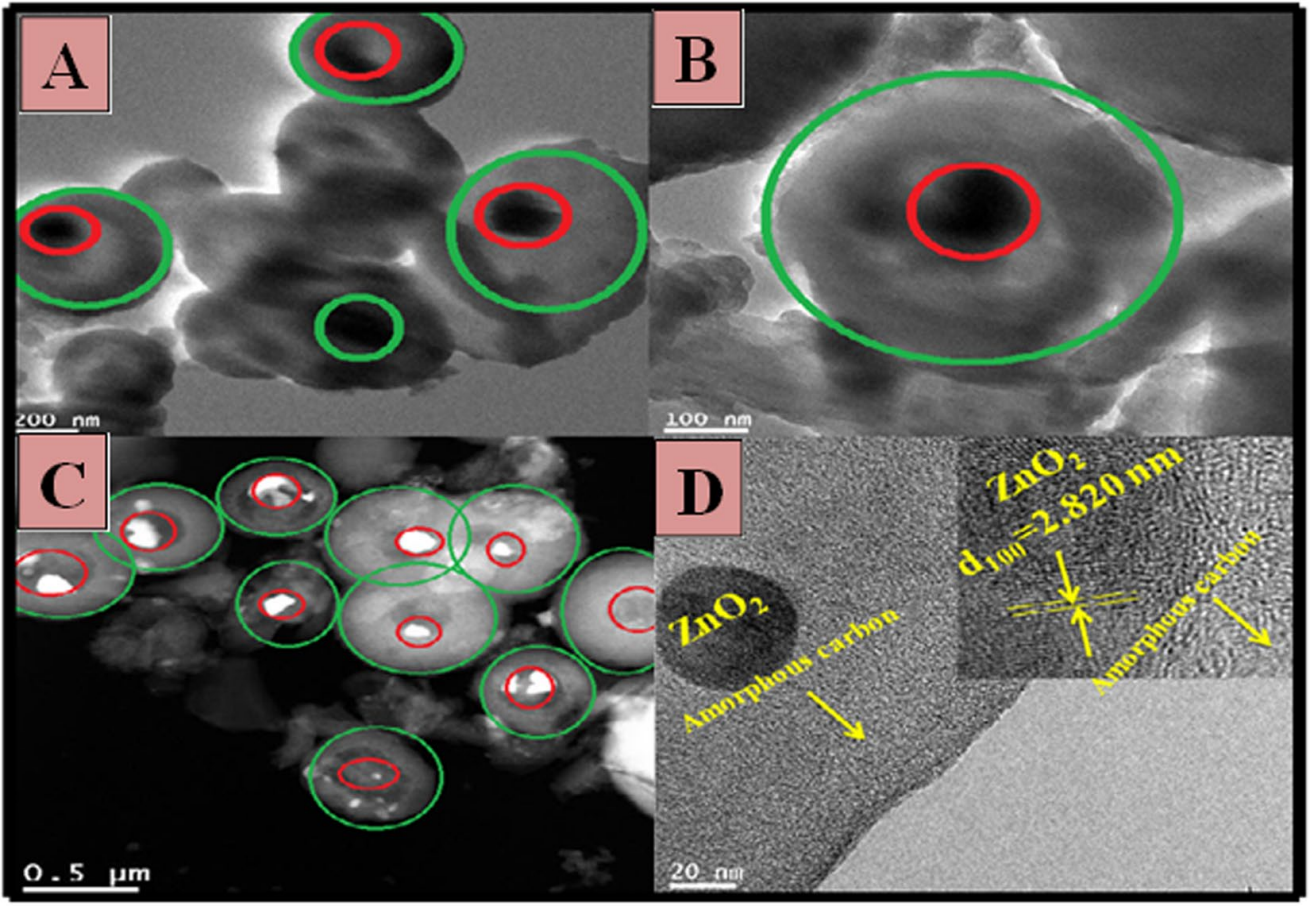
(**A–C**) TEM images, (**D**) High resolution TEM image for the obtained core@shell (ZnO@C) microspheres after carbonized at 700 °C for 2 h in nitrogen atmosphere.

Additionally, the prepared microspheres were investigated by TEM analysis (Fig. 3(A–D)). The perfect interconnection between core (ZnO) and shell (carbon) can be seen (Fig. 3(A–C)). The HR-TEM image (Fig. 3(D) of the investigated microsphere clearly evidenced the lattice plane with 0.282 nm inter planar distance (d-spacing) associated to (100) plane of ZnO with the outer amorphous region ascribed to the graphitic carbon (marked by an arrows). A similar morphology was also observed by other authors^25, 26^. The mean diameter of the cores and shells of microspheres are 100 and 150 nm, respectively.

**Figure 4:**
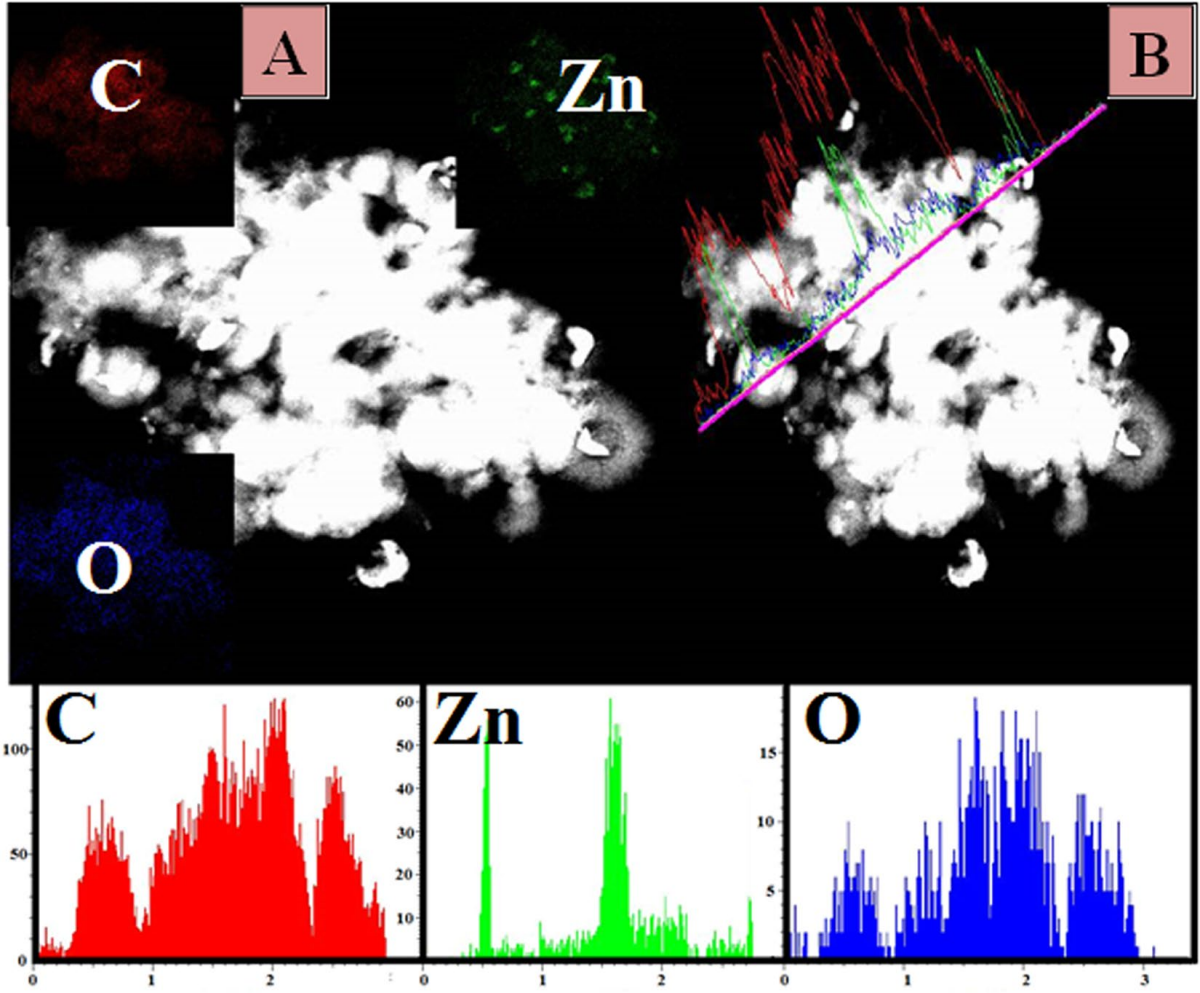
(**A**) Elemental maping, (**B**) Line TEM EDX analysis for the obtained core@shell (ZnO@C) microspheres after carbonized at 700 °C for 2 h in nitrogen atmosphere.

The presence and distribution of Carbon, Oxygen and Zinc elements were also recognized by TEM mapping, the results are shown in Fig. 4(A). The presence of Zn is revealed along with C and O. The carbon corresponding micrographs show that C is evenly distributed on the surface of Zn. However, Zinc and some parts of Oxygen are concentrated in a specific area in the form of ZnO@C (core @shell) microspheres. Further evidence is provided by TEM line EDX, as shown in the main image (Fig. 4(B)), carbon has high intensity with a wide distribution compared to the metallic counterpart. Moreover, as shown in the concentration profiles, Zn, O, and C were detected along with the selected line. Overall, it can be claimed that the observed metallic counterparts (Fig. 3(A–C) marked by red circles) are sheathed in a carbon shells (Fig. 3(A–C) marked by green circles).

In the direct methanol fuel cells, the conversion of methanol to carbon dioxide (${\boldsymbol{C}}{{\boldsymbol{H}}}_{3}{\boldsymbol{OH}}+{{\boldsymbol{H}}}_{2}{\boldsymbol{O}}={\boldsymbol{C}}{{\boldsymbol{O}}}_{2}+{\bf{6}}{\boldsymbol{H}}+{{\bf{6}}}^{-{\boldsymbol{e}}}$ **)** is well-thought-out to be the combination of adsorption and electrochemical reaction on the anodic surface. Therefore, due to the well documented adsorption capability of carbon, it has been combined in many lately documented electrocatalytic materials^4, 5, 27^.

**Figure 5:**
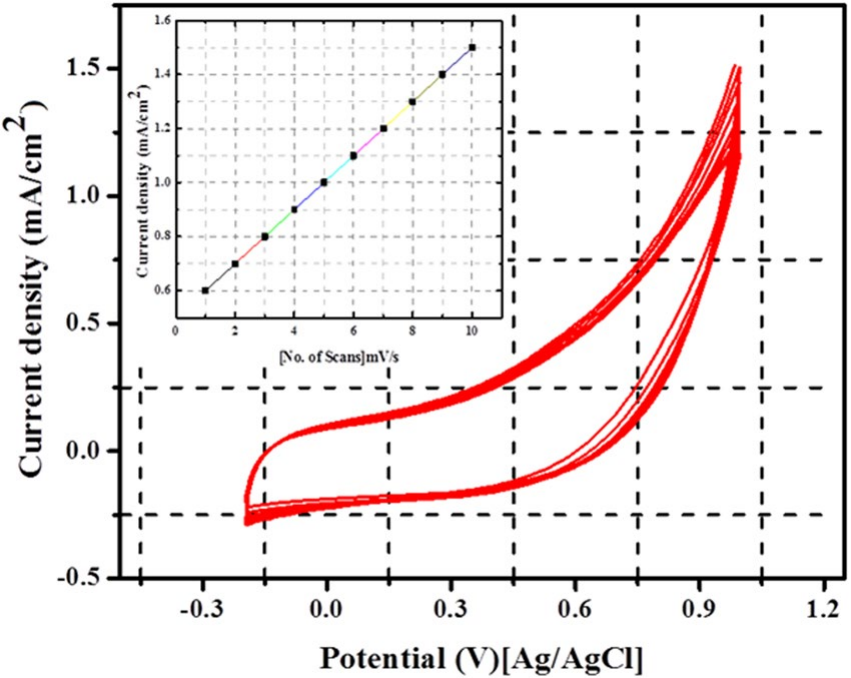
Cyclic voltammograms for the obtained core@shell (ZnO@C) microspheres in 1 M KOH at the scan rate of 50 mV/s; inset image: dependency of the current density on the number of CV cycles (1 to 10 cycles).

Likened to nickel, pristine non-precious metals have low electrocatalytic activity because nickel can be electrochemically stimulated by forming active compounds (NiOOH) on the surface which generate good electrochemical activity. Cyclic voltammetry study was carried out to investigate the electrocatalytic activity of the synthesized ZnO@C electrode in the potential range of −200 mV/s to 1000 mV/s (vs. Ag/AgCl reference electrode) at a scan rate of 50 mV/s in 1.0 M KOH solution (absence of methanol), the results are displayed in Fig. 5. Besides the high electrocatalytic activity of Pt-based electrodes compared to the non-precious materials, dispensing of surface activation is another important feature. It is well known that nickel-based materials are the most widely used non precious electrocatalysts. It is known that the surface of the Ni-based electrocatalysts should be activated by formation of NiOOH layer which can be achieved by multiple CV analysis in presence of KOH solution. In other words, the original electronic structure of the precious metal (Pt) is highly adequate to catalyze the methanol oxidation reaction. As shown in Fig. 5, no OOH^−^ formation peak appears in the cycles which indicate that there is no change in the surface structure upon sweeping in the alkaline media. Generally, during the activation process of the nickel-based materials, in the voltammogram, two regions are observed; the first region is in the negative potential side comprising an anodic peak representing the oxidation of nickel according to the reaction^28^:

1 $${\boldsymbol{Ni}}+2{\boldsymbol{O}}{{\boldsymbol{H}}}^{-}\leftrightarrow {\boldsymbol{Ni}}{({\boldsymbol{OH}})}_{2}+2{\boldsymbol{e}}$$

The corresponding peak is usually very small in the first cycle and disappears in the subsequent ones^28–30^. The second region is observed in the positive potential side, this peak is always strong and related to the oxidation of Ni(OH)_2_ to NiOOH in accordance with this reaction^30–32^:

2 $${\boldsymbol{Ni}}{({\boldsymbol{OH}})}_{2}+{\boldsymbol{O}}{{\boldsymbol{H}}}^{-}\leftrightarrow {\boldsymbol{NiOOH}}+{{\boldsymbol{H}}}_{2}{\boldsymbol{O}}+{\boldsymbol{e}}$$

Increasing the number of potential sweeps results in a progressive increase of the current density values of the cathodic peak because of the entry of OH^−^ into the Ni(OH)_2_ surface layer, which leads to the progressive formation of a thicker NiOOH layer corresponding to the Ni(OH)_2_/NiOOH transition^28^. As can be observed in Fig. 5, the proposed electrocatalyst does not show any redox peaks which indicated that no ZnOOH layer was formed.

The increase in the current density at high potential can be assigned to the water oxidation reaction. The inset image of Fig. 5 shows a linear dependence of the anodic current on the potential sweeps from 1 to 10 cycles with a correlation coefficient of 0.9989 which can be attributed to surface physical activation^4, 5^.

**Figure 6:**
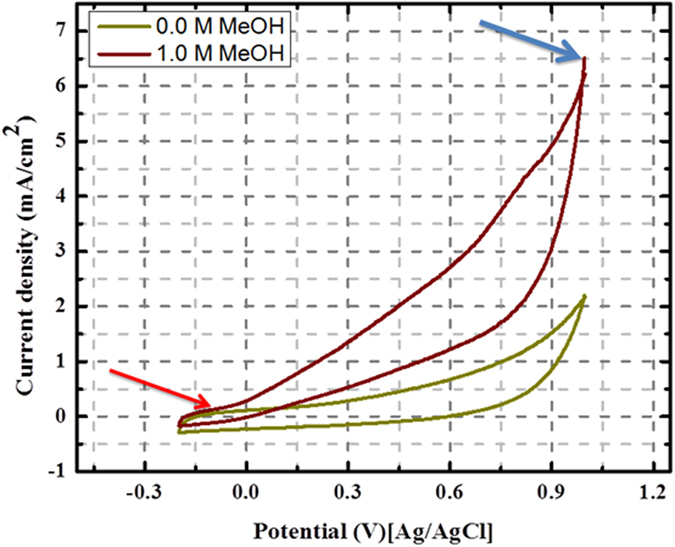
Cyclic voltammograms for the obtained core@shell (ZnO@C) microspheres in absence and presence of 1 M Methanol in 1 M KOH solution at the scan rate of 50 mV/s.

Figure 6 presents the typical CV of the synthesized core@shell structured ZnO@C electrode measured in absence and presence of 1.0 M methanol in 1.0 M KOH solution at a scan rate of 50 mV/s. CV curves (Fig. 6) specify operative methanol oxidation with current density of 6.5 mA/cm^2^ at 100 mV (marked by blue arrow). However, the most important finding is the observed onset potential. As shown, a negative value (−90 mV vs. Ag/AgCl) was obtained (marked by red arrow). Moreover, no redox peaks were observed, indicating that presented electrode is fairly stable in alkaline medium^33^. Remarkably, the results demonstrate that the presented electrode owned fairly good catalytic activity toward methanol electro-oxidation in term of current density and onset potential. It is likely that the methanol electrooxidation takes place on the surface of electrode. The onset potential is very useful for the investigation of electrocatalytic activity and can be utilized to assess the effectiveness of electrocatalyst. Basically onset potential is the point at which a reaction product is formed. In direct methanol fuel cells, more negative onset potential for the anodic reaction specifies high catalytic activity.

**Figure 7:**
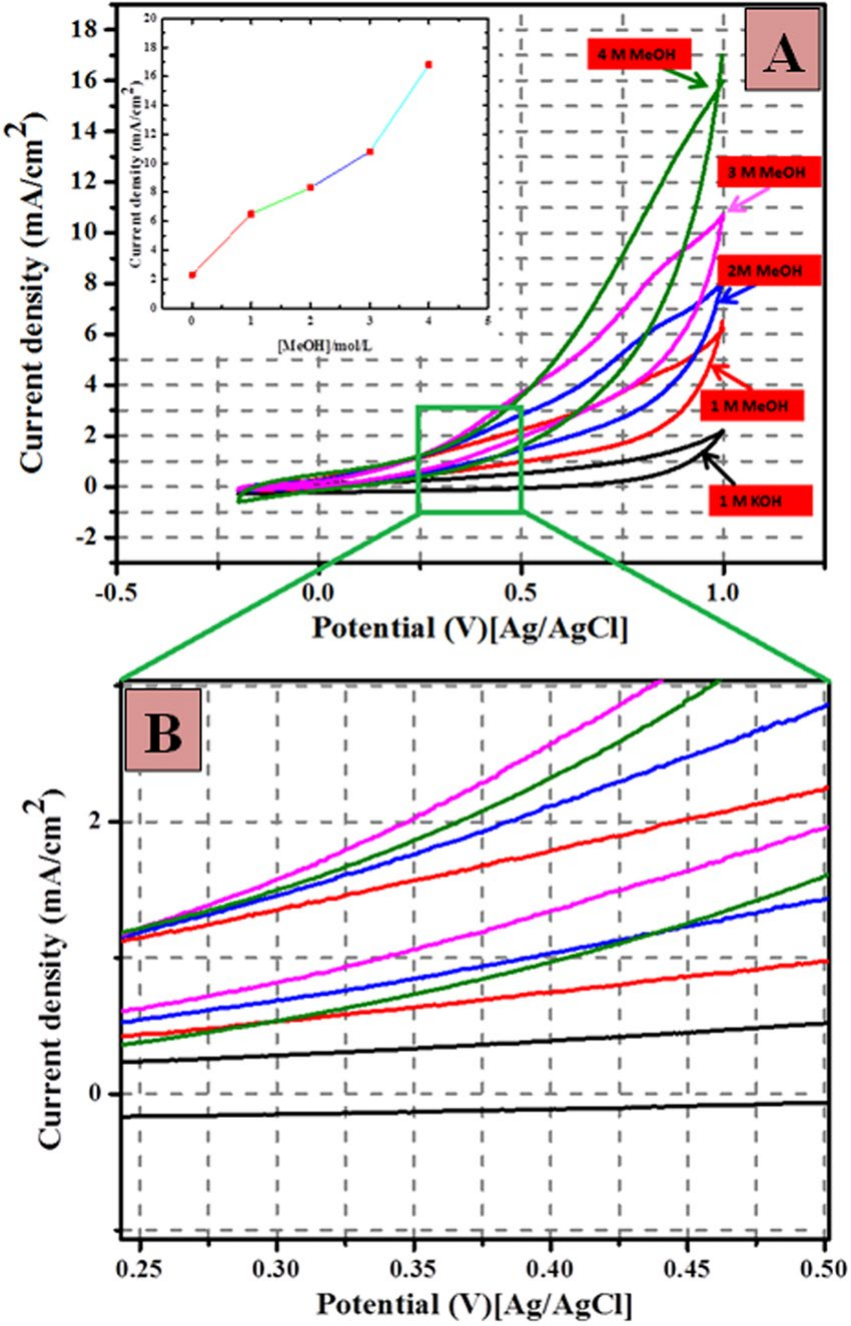
Typical cyclic voltammogram for the obtained core@shell (ZnO@C) carbon spheres in 1 M KOH solution in the presence of different concentration of methanol; inset image: dependency of the current density on the concentration of methanol in solution and high megnification (**B**) to show the onset potential at the scan rate 50 mV/s at 25 °C.

Figure 7 shows the impact of methanol concentration on the electrocatalytic performance of synthesized core@shell structured ZnO@C electrode for methanol electrooxidation. The concentration of methanol was changed while the concentration of KOH was kept constant (1.0 M) throughout the experiments. It can be observed from Fig. 7(A), the anodic current increases with increasing the methanol concentration. Remarkably, oxidation current densities increased drastically with increasing the methanol concentration from 0 to 4 M at very low potential (0.25 V (vs. Ag/AgCl)). Figure 7(B) stated a wide scale for the marked area, as shown the acquired onset potential is very low compared to many reported materials^8, 34, 35^. The inset image of Fig. 7(A) illustrates an almost direct relationship between the anodic current density and the concentration of methanol with a correlation coefficient of 0.979. The results further confirm that methanol is involved in oxidation and suggesting that the increase of fuel amount can improve the current density.

A proposed mechanism for the electro oxidation of methanol on ZnO@C based on previous studies^36, 37^ as follow: In the first three steps, methanol was adsorbed on ZnO@C surface and protons are released.

$$\begin{array}{c}{\boldsymbol{Step}}1,\,{\boldsymbol{C}}{{\boldsymbol{H}}}_{3}{\boldsymbol{OH}}+{\boldsymbol{ZnO}}@{\boldsymbol{C}}\leftrightarrow ({\boldsymbol{ZnO}}@{\boldsymbol{C}}){\boldsymbol{C}}{{\boldsymbol{H}}}_{2}{\boldsymbol{OH}}+{{\boldsymbol{H}}}^{+}+{\boldsymbol{e}}\\ {\boldsymbol{Step}}2,\,({\boldsymbol{ZnO}}@{\boldsymbol{C}}){\boldsymbol{C}}{{\boldsymbol{H}}}_{2}{\boldsymbol{OH}}+({\boldsymbol{ZnO}}@{\boldsymbol{C}})\to {({\boldsymbol{ZnO}}@{\boldsymbol{C}})}_{2}{\boldsymbol{CHOH}}+{{\boldsymbol{H}}}^{+}+{\boldsymbol{e}}\\ {\boldsymbol{Step}}3,\,{({\boldsymbol{ZnO}}@{\boldsymbol{C}})}_{2}{\boldsymbol{CHOH}}+{\boldsymbol{ZnO}}@{\boldsymbol{C}}\to {({\boldsymbol{ZnO}}@{\boldsymbol{C}})}_{3}{\boldsymbol{COH}}+{{\boldsymbol{H}}}^{+}+{\boldsymbol{e}}\\ {\boldsymbol{Step}}4,\,{({\boldsymbol{ZnO}}@{\boldsymbol{C}})}_{3}{\boldsymbol{COH}}\to ({\boldsymbol{ZnO}}@{\boldsymbol{C}}){\boldsymbol{CO}}+2({\boldsymbol{ZnO}}@{\boldsymbol{C}})+{{\boldsymbol{H}}}^{+}+{\boldsymbol{e}}\end{array}$$

In the fourth step (*ZnO*@*C*)_3_ *COH* decomposes to produce (*ZnO*@*C*)*CO* and protons. Finally, carbon monoxide is oxidized by the hydroxyl group^36^.

**Figure 8:**
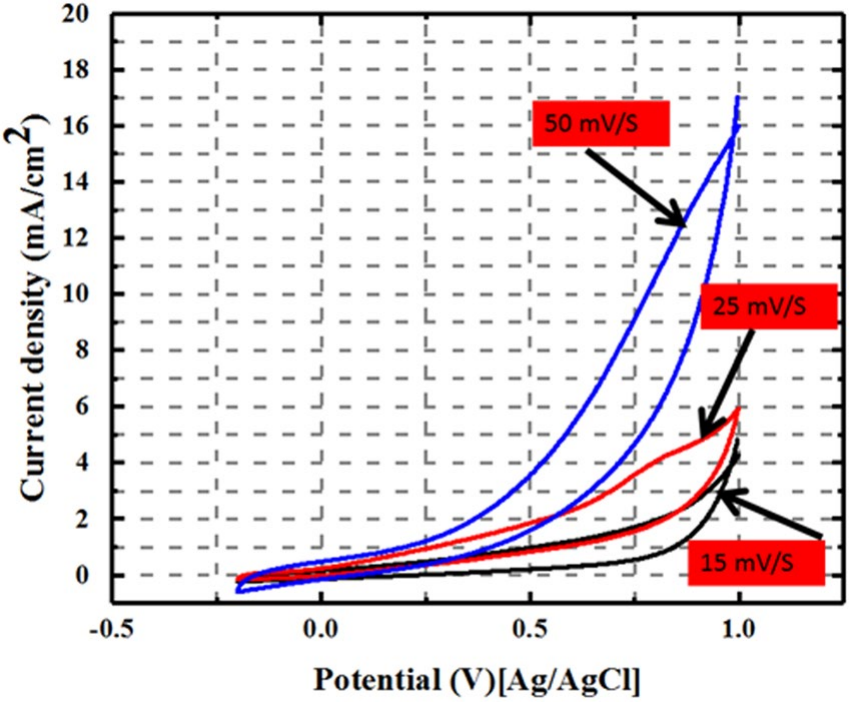
Cyclic voltammograms for the obtained core@shell (ZnO@C) carbon spheres in 1 M KOH soution in the presence of 4 M methanol at various scan rates.

The effect of scan rate on electrocatalytic activity is helpful diagnostic tool to investigate the rate controlling step. Figure 8 displays the effect of the scan rate on the electrocatalytic activity of the synthesized core@shell structured ZnO@C electrode at different scan rates in 4 M methanol. It can be noticed that the anodic current density increases with increasing the scan rate from 15 to 50 mV/s. This result indicates the rapid ionic transformation on the surface of the electrode during the electrooxidation of methanol and reveals that the oxidation process is not limited by diffusion.

## Conclusions

Spent coffee grounds can be efficiently utilized to synthesize Core@shell (ZnO@C) microspheres as a new, cheap and readily available natural source of carbon. The introduced ZnO@C shows satisfactory current density toward methanol oxidation. Moreover, the current density increased with increasing the fuel concentration. A notable low onset potential reveal its impressive electrocatalytic activity, it is rational to conclude that the core@shell (ZnO@C) microspheres with good electrocatalytic activity can serve as an auspicious anode material for fuel cells application.

## Experimental

### Materials and Preparation of ZnO@C microspheres

Spent coffee grounds were obtained from university coffee shop, dried at 50 °C for 6 h and powdered after received. Zinc Chloride (98.0%) was obtained from Samchun Pure Chemicals, South Korea. All the reagents were of analytical grade and utilized without further purification.

A 5.0 g of spent coffee grounds was taken in a reaction flask and mixed with a 1:1 weight ratio of ZnCl_2_ (which was previously dissolved in deionized water) and left over night at room temperature. Finally, the mixture was filtered and washed 6 times with deionized water and then the solid cake was dried in a vacuum oven at 60 °C for 12 h. After drying the coffee grounds + ZnCl_2_ mixture was carbonized in a tube furnace under a flow of N_2_ gas at a heating rate of 2 °C/min up to 700 °C, and held at this temperature for 2 h.

### Characterization and electrochemical measurement

Information about the phase structure and crystallinity was determined by powder X-ray diffraction spectrum (XRD, Rigaku Japan,) with Cu-Kα (λ = 1.54056 Å) radiation operating at 45 kV and 100 mA over a range of 2θ angle from 10° to 80°, scanning at a rate of 4◦/min. The morphology of the as-prepared sample was examined with field-emission scanning electron microscopy (FESEM, Hitachi S-7400, Japan). TEM images were observed by JEOL JEM-2200FS transmission electron microscope (TEM) operating at 200 kV (JEOL, Japan). Moreover, the electrochemical oxidation of the methanol were carried out in a conventional three electrode electrochemical cell (VersaSTAT 4, USA) with a glassy carbon electrode as working electrode. Saturated Ag/AgCl and Pt were utilized as a reference and counter electrodes, respectively. Fabrication of the working electrode was carried out by mixing 2.0 mg of the as-synthesized electrocatalyst, 400 μL of isopropanol and 20 μL of Nafion solution (5 wt. %). The slurry was sonicated for a minimum of 30 min to form homogeneous ink. Then 15 μL ultrasonically dispersed catalyst ink was loaded onto the active area of the glassy carbon electrode which was then subjected to drying process at 80 °C for 30 min.
